# Supplementary material for: Modeling glioblastoma heterogeneity as a dynamic network of cell states
Source: Mol Syst Biol. 2021 Sep 16;17(9):e10105. doi: 10.15252/msb.202010105 (PMC8444284; doi:10.15252/msb.202010105)
Supplement: Supplementary file 6 — Source Data for Figure 5 [file MSB-17-e10105-s004.zip › Figure5A_sourcedata/GSEA_3071/nosigdb_stateV.GseaPreranked.1622795159954/SEGERMAN_MULTITHERAPY_RESISTANCE_FIG5A_PMID_27974212.html]

Details for gene set SEGERMAN\_MULTITHERAPY\_RESISTANCE\_FIG5A\_PMID\_27974212[GSEA]

|  || Dataset | state43071 |
| Phenotype | NoPhenotypeAvailable |
| Upregulated in class | na\_neg |
| GeneSet | SEGERMAN\_MULTITHERAPY\_RESISTANCE\_FIG5A\_PMID\_27974212 |
| Enrichment Score (ES) | -0.7455917 |
| Normalized Enrichment Score (NES) | -2.9347825 |
| Nominal p-value | 0.0 |
| FDR q-value | 0.0 |
| FWER p-Value | 0.0 |
Table: GSEA Results Summary

  

Fig 1: Enrichment plot: SEGERMAN\_MULTITHERAPY\_RESISTANCE\_FIG5A\_PMID\_27974212      
 Profile of the Running ES Score & Positions of GeneSet Members on the Rank Ordered List

  

| PROBE | GENE SYMBOL | GENE\_TITLE | RANK IN GENE LIST | RANK METRIC SCORE | RUNNING ES | CORE ENRICHMENT || 1 | SMPD1 |  |  | 2240 | 0.358 | -0.4098 | No |
| 2 | SLC25A43 |  |  | 3479 | -0.296 | -0.6322 | No |
| 3 | TBX15 |  |  | 3547 | -0.307 | -0.6320 | No |
| 4 | TRIM16 |  |  | 4147 | -0.422 | -0.7279 | Yes |
| 5 | OSTF1 |  |  | 4231 | -0.449 | -0.7248 | Yes |
| 6 | FSTL1 |  |  | 4262 | -0.460 | -0.7112 | Yes |
| 7 | BLVRB |  |  | 4371 | -0.491 | -0.7111 | Yes |
| 8 | IGDCC4 |  |  | 4411 | -0.504 | -0.6973 | Yes |
| 9 | TMBIM1 |  |  | 4427 | -0.512 | -0.6787 | Yes |
| 10 | SCPEP1 |  |  | 4589 | -0.579 | -0.6850 | Yes |
| 11 | LOXL1 |  |  | 4607 | -0.593 | -0.6633 | Yes |
| 12 | SERPINB1 |  |  | 4659 | -0.623 | -0.6468 | Yes |
| 13 | MVP |  |  | 4760 | -0.686 | -0.6370 | Yes |
| 14 | PPIC |  |  | 4802 | -0.716 | -0.6147 | Yes |
| 15 | CLMP |  |  | 5008 | -0.948 | -0.6138 | Yes |
| 16 | QSOX1 |  |  | 5018 | -0.968 | -0.5750 | Yes |
| 17 | COPZ2 |  |  | 5069 | -1.073 | -0.5394 | Yes |
| 18 | SHC1 |  |  | 5094 | -1.133 | -0.4965 | Yes |
| 19 | MSRB3 |  |  | 5254 | -2.046 | -0.4408 | Yes |
| 20 | DAB2 |  |  | 5261 | -2.217 | -0.3490 | Yes |
| 21 | AHNAK |  |  | 5268 | -2.309 | -0.2533 | Yes |
| 22 | FN1 |  |  | 5287 | -2.866 | -0.1365 | Yes |
| 23 | EDIL3 |  |  | 5290 | -3.285 | 0.0009 | Yes |
Table: GSEA details [plain text format]

  

Fig 2: SEGERMAN\_MULTITHERAPY\_RESISTANCE\_FIG5A\_PMID\_27974212: Random ES distribution      
 Gene set null distribution of ES for **SEGERMAN\_MULTITHERAPY\_RESISTANCE\_FIG5A\_PMID\_27974212**
